# Anthropogenic oligotrophication via liming: Long-term phosphorus trends in acidified, limed, and neutral reference lakes in Sweden

**DOI:** 10.1007/s13280-014-0573-0

**Published:** 2014-11-15

**Authors:** Qian Hu, Brian J. Huser

**Affiliations:** 1 Waterways Centre for Freshwater Management, University of Canterbury, Private Bag 4800, Christchurch, New Zealand; 2 Department of Aquatic Sciences and Assessment, Swedish University of Agricultural Sciences, Box 7050, 75007 Uppsala, Sweden

**Keywords:** Productivity, Liming, Sediment, Phosphorus, Metal precipitation, Acidified lakes

## Abstract

Restoration of acidified lakes by liming does not, in many cases, improve productivity to a pre-acidified state. We hypothesize that the poor recovery detected in many of these lakes is due to constrained in-lake phosphorous (P) cycling caused by enhanced precipitation of metals in higher pH, limed waters. Long-term (1990–2012) data for 65 limed, circum-neutral (pH 6–8), and acidified lakes in Sweden were analyzed to determine trends for P and potential drivers of these trends. Limed lakes not only had lower mean values and stronger decreasing trends for total P than non-limed lakes, but they also had the highest percentage of decreasing trends (85 %). A P release factor (Hypolimnetic P/Epilimnetic P) was developed to elucidate differences in internal P cycling between lake groups. Consistently, lower P release factors in limed lakes show limitation of internal P cycling during summer months that may be a factor limiting P bioavailability and thus productivity of these systems.

**Electronic supplementary material:**

The online version of this article (doi:10.1007/s13280-014-0573-0) contains supplementary material, which is available to authorized users.

## Introduction

Acidification of lakes in Scandinavia and other areas of the world is not a new topic, but issues still remain with how to restore these systems (Clair and Hindar 2005). Since the late 1960s, researchers have shown high transparency, the appearance of plankton communities associated with nutrient-starved systems, and extinction of local fish species in acidified lakes (Jansson et al. 1986; Dickson 1988). In Sweden, a large scale lake liming program was implemented during the late 1970s to protect biodiversity and fish in acidified lakes (Appelberg and Svenson 2001). Lime treatments have resulted in considerably improved water chemistry and biological diversity compared to acidified lakes (Henrikson and Brodin 1995), but lake productivity has generally not improved to pre-acidified conditions (Weatherley 1988; Clair and Hindar 2005). Based on ISELAW (integrated studies of the effects of liming acidified waters) monitoring from 1989 to 1998, reduced productivity in limed lakes compared to circum-neutral reference lakes was indicated by low total phosphorus (P) concentrations, increased total nitrogen concentrations, impoverishment of benthic invertebrates, and reduced species diversity of fish (Appelberg and Svenson 2001).

P is generally the limiting nutrient in most lakes (Schindler and Fee 1974), meaning any reduction or increase will directly change in-lake productivity. Studies on lake liming have shown somewhat contradictory results with respect to P, with both increased and reduced epilimnetic total P (TP) concentrations (Broberg 1987), or no difference in TP concentration between limed and non-limed lakes (Wilander et al. 1995). However, for a number of reasons, limed lakes could be expected to have lower TP concentrations compared to non-limed lakes over the long term. First, limed lakes will most likely have acidified watersheds, from which lower amounts of P are transported to lakes (Persson and Broberg 1985; Jansson et al. 1986; Wilander et al. 1995). Second, precipitation of watershed-derived metals (soluble, organically bound, and those liberated from organic matter via photo-oxidation) is enhanced in limed waters (Borg et al. 2001; Wällstedt and Borg 2005) due to improved conditions for photo-oxidation and oxy-hydroxide formation (Andersen and Pempkowiak 1999). Increased metal precipitation increases P sedimentation and reduces internal P cycling in lakes because aluminum (Al) complexes can bind phosphate strongly and co-precipitate P (Ulrich and Pöthig 2000; Kopacek et al. 2007). In addition, particles coated with Al, iron (Fe), and manganese (Mn) have great affinity for phosphorous (Froelich 1988). Finally, accumulation of both watershed-derived metals, along with those found in liming materials (both Fe and Al), can lead to long-term accumulation of these metals in the sediment (Huser and Rydin 2005; Wällstedt and Borg 2005). The increased potential for P binding in both water and sediment may be a factor behind why many limed lakes do not recover to pre-acidified nutrient conditions.

In this study, long-term (1990–2012) data for limed, acidified, and circum-neutral (pH 6–8, hereafter neutral) reference lakes in Sweden were investigated. Mean epilimnetic concentrations and long-term trends were determined for total P (TP) and compared between the three groups of lakes (acidified, limed, and neutral) to determine relative differences as well as changes over time. A P release factor (hypolimnetic P/epilimnetic P) was also developed to elucidate the potential control internal P cycling has on P availability in the study lakes.

## Materials and methods

### Data collection and preparation

Land-use, climatic factors, and geomorphology (i.e., depth/lake area) of the lake groups were generally similar, varying by less than a factor of 2 (Table 1). Land-use was calculated using CORINE land cover data that were aggregated into 18 classes. Watershed boundaries were delineated using elevation data in a 50 meter grid with a maximum standard error of 2.5 m. Land-use was dominated by forest (62.8–72 % of total area), mean depth and lake area ranged from 3.7–6.9 m to 0.54–1.1 km^2^, and annual mean temperature and precipitation ranged from 5.9–6.8 °C and 765–867 mm, respectively.

**Table 1:** Mean land-use (% area), climate (annual means), and geomorphology of the study lakes by lake group

	Open water	Wetland	Forest	Urban	Agriculture	Logged	Other	Air temp. (°C)	Precipitation (mm)	Mean depth (m)	Area (km^2^)	Lake area/watershed area
Acidified	12.1	8.1	72.0	0.0	1.6	6.2	0	6.8	867	3.7	0.54	0.12
Limed	13.4	11.1	62.8	0.2	1.5	8.1	2.9	5.9	849	6.9	1.1	0.10
Neutral	15.5	5.3	64.5	0.1	2.8	9.6	2.1	6.1	765	6.1	1.1	0.14

**Fig. 1:**
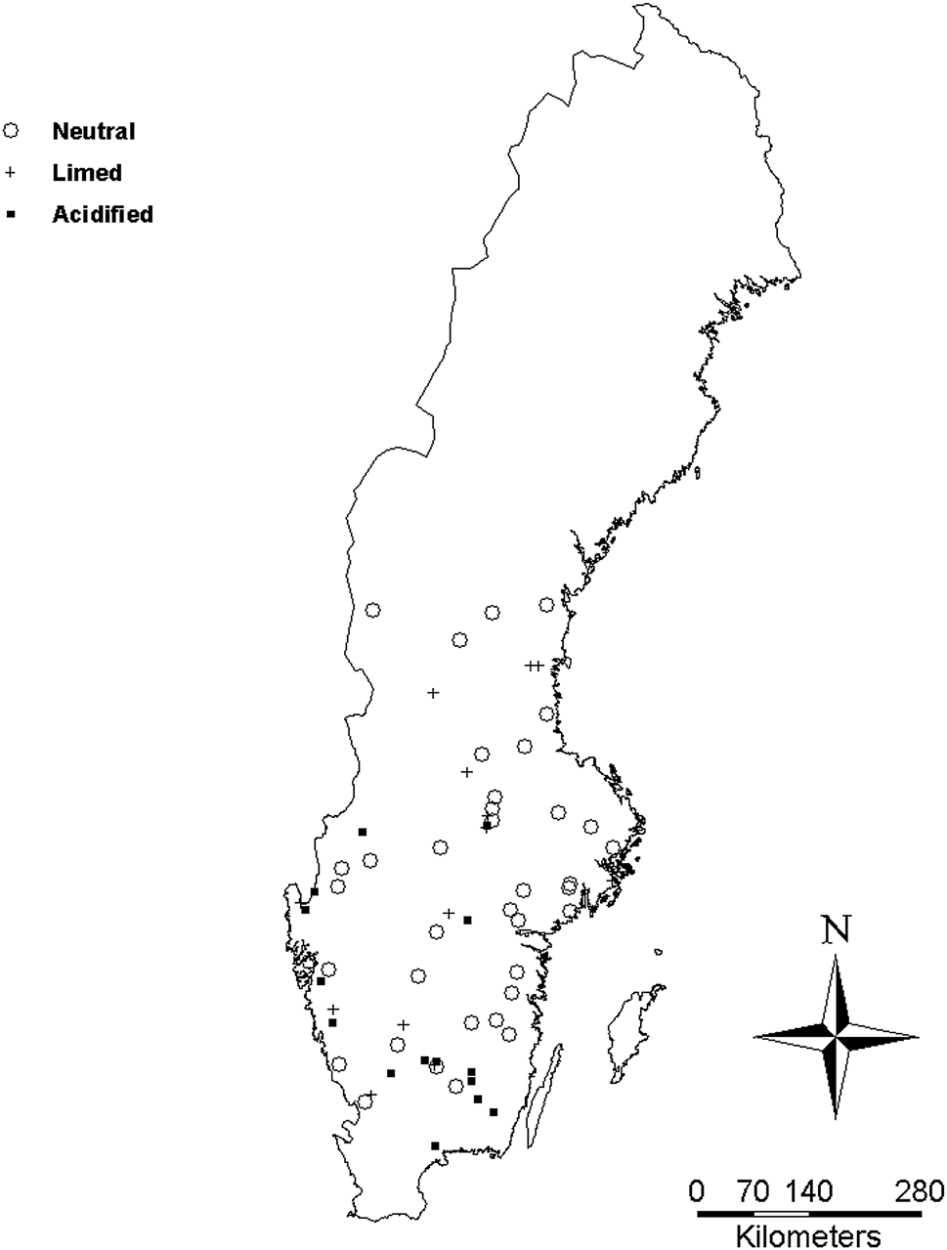
Geographical location of lakes included in the study

Water chemistry data were collected from the database managed by the Swedish University of Agriculture Sciences, Department of Aquatic Sciences and Assessment (http://info1.ma.slu.se) and were compiled using JMP statistical software (SAS, version 10.0.0). Both limed and non-limed lakes were found within the database. For the non-limed lake groups, acidified lakes had to be acidified according to the Model of Acidification of Groundwater In Catchments (MAGIC) model (i.e., decrease in pH ≥ 0.4 pH units between 1850 and 2012, Cosby et al. 1985) with a median pH below 5.5. Neutral lakes were defined as lakes with a median pH between 6.0 and 8.0; these lakes were also compared to the MAGIC database to assure that they were not anthropogenically acidified. Lakes in the Arctic region were excluded due to regional climatic differences and the high occurrence of naturally acidic lakes. 69 lakes, from a total of 99 lakes (8 slightly acidic, pH 5.5–6.0, and 22 from the arctic region omitted), were included in the study (Fig. 1), and 8219 surface samples, representing the epilimnion, were used to construct the main dataset. 1940 hypolimnetic samples (i.e., the deepest sample in lakes where multiple water column samples were collected) from 25 stratified lakes were analyzed in the study to determine potential changes to internal lake cycling of P over the study period. Land-use, geomorphological, and climatic data for the lakes, by group, are shown in Table 1.

TP concentration data from surface water samples were arranged into seasonal time series. From each lake, one TP concentration value per season was chosen and arranged chronically from 1990 to 2012. Chronologically, in this case, means water samples representing winter come first, followed then by spring, summer and autumn samples (Electronic Supplementary Material, Table 10.1007/s13280-014-0573-0). The number of samples collected within each season was not always equal. Thus, to avoid differences in statistical variance within each season, only one sample per season per lake was chosen for analysis. In the case of multiple samples within a season, the sample chosen for analysis should be the most representative one from that season. Accordingly, the late winter sample represented the most stable winter status, whereas the spring sample just after ice breakup and lake turnover was most representative. Late summer samples when lakes were strongly stratified were preferred, whereas autumn samples representing mixed conditions after lake turnover were selected (Electronic Supplementary Material, Table 10.1007/s13280-014-0573-0). Based on these seasonal priorities, samples collected in the first ranked month were chosen. If no sample was collected in that month, a sample in the second ranked month was chosen, and so on. If two or more samples (usually no more than two) were taken in a same month, the one with the sampling date closest to the middle of that month was prioritized. Then a seasonal time series dataset of surface water TP concentration was created.

Time series shorter than 10 years were excluded because trend analysis requires seasonal time series longer than 10 years for statistically valid results (Loftis et al. 1991). To compare different lake groups, the same time period (1990–2012) was chosen. Outliers, defined as data beyond the 3rd quartiles by 1.5 interquartile ranges, and times series containing non-continuous TP data, were removed. The 69 lakes were again tested against the MAGIC database that estimates whether an acidic lake is naturally acidic or anthropogenically acidified (Moldan et al. 2013) and verified using the modeling results of Valinia et al. (2014). Four lakes in the dataset were naturally acidic and not included in the main analysis because the number of lakes was too low to create a naturally acidic lake group.

### Phosphorous release factor

For lakes with profile samples collected throughout the water column (stratified lakes), the ratio of hypolimnetic TP concentration to epilimnetic TP concentration was used as a phosphorous release factor (PRF). If the PRF was equal to or less than one, it indicated a tendency for increased retention (or limited release) of P from the bottom sediment, likely due to elevated sorption capacity provided by P binding metals (Huser and Rydin 2005). Seasonal time series of PRF-values (1990–2011) were created for each lake using the method used for epilimnetic TP seasonal time series. After excluding time series shorter than 10 years and those with non-continuous data, 23 PRF seasonal time series remained (i.e., 11 limed, 5 acidified, 7 neutral lakes).

### Data analysis

Statistic analyses were conducted in R (version i386 2.15.0). For the epilimnetic TP seasonal time series, mean TP concentrations between 1990 and 2012 for each lake were calculated by averaging the mean seasonal TP. Box-plots of the mean epilimnetic TP concentrations by lake group (acidified, limed, neutral) were made, and outliers (data beyond the 3rd quartiles by 1.5 interquartile ranges) were removed. The Shapiro–Wilk’s test was used to check data normality, and mean TP concentrations for all groups were normally distributed. Differences in distribution variances were then determined using the *F* test. The Student’s *T* test was used to compare TP concentration between groups with equal variance, whereas the Welch Two Sample test was employed when unequal variance was found.

Seasonal time series for epilimnetic TP were analyzed using the Seasonal-Kendall Statistic Trend test, in this case, a Visual Basic program in Microsoft Excel for multivariate Mann–Kendall tests of monotonic trends (Loftis et al. 1991). Significant monotonic trends (*p* ≤ 0.05) for epilimnetic TP concentration were determined, and Theil slopes were calculated as estimates of the TP trend slopes (absolute change per year) for each lake. Dividing each slope value by the mean epilimnetic TP concentration for the corresponding lake resulted in normalized Theil slopes. The normalized Theil slopes were then subjected to group comparison. After normality check (all groups were normally distributed), either the Student’s *T* test (equal variance) or the Welch Two Sample test (unequal variance) was used.

To define the annual sediment P release potential in each lake (annual PRF-value index), the percentage of PRF-values less than or equal to one for each year was calculated. For each lake group, annual release potential was calculated from the seasonal PRF time series. Annual release potential from 1990 to 2011 for the three lake groups was also analyzed using the Mann–Kendall Trend test (described above). Lastly, summer PRF-values from the refined 23 PRF seasonal time series were separated to compare among lake groups. Distributions of the summer PRF-values of the three lake groups were extremely skewed and transformation to normality could not be satisfied. Thus, Levene’s test was used to exam variance difference among groups, and Kolmogorov–Smirnov test was employed to compare the distributions of PRF-values due to unequal variance among lake groups.

### Data quality assessment

Machine calibration drift caused an average overestimation of TP concentration of 1.2 ± 0.2 μg L^−1^ (95 % confidential intervals) from January 1991 through June 1996 (Sonesten and Engblom 2001). In our study, we adjusted the TP dataset during this period by subtracting 1.2 µg L^−1^ from all TP concentrations during this period, and this “calibrated” TP dataset was analyzed in parallel with the original TP data. All individual lake datasets were also visually inspected for any abnormalities due to potential drift. Results from both the original and calibrated dataset were similar (both visually and statistically), and thus only the results from the original data are presented.

## Results

**Fig. 2:**
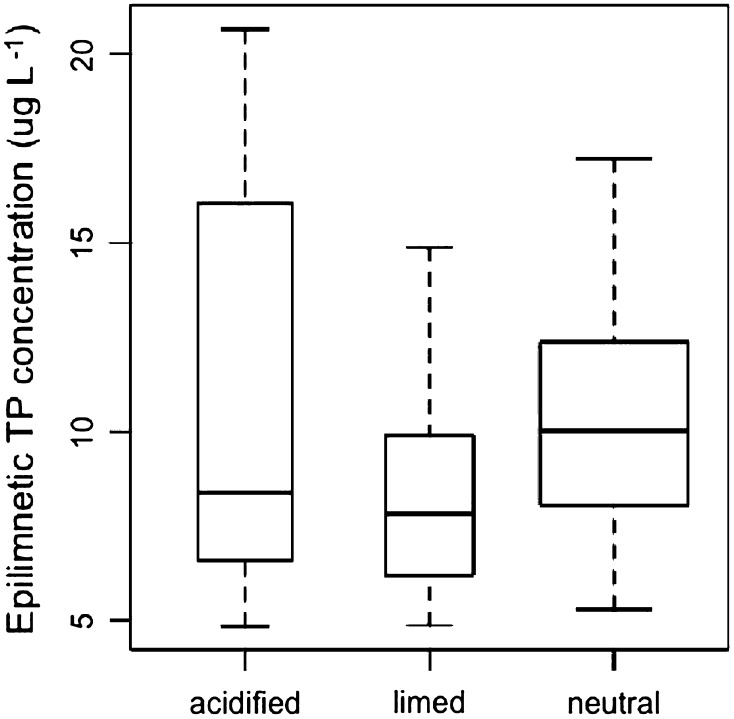
Box-plot of epilimnetic TP by lake group (1990–2012). In *each box*, the *central line* is the median value, the *upper* and *lower limits* of *each box* are the lower and upper quartiles (25 and 75 %), and the *whiskers* extend 1.5 times the interquartile range

Mean, epilimnetic TP concentrations for each group were generally low (oligotrophic), and the neutral lake group had the highest mean TP concentration of 12.1 μg L^−1^ (median = 10.0 μg L^−1^, Fig. 2). TP concentrations for the limed lake group (mean = 8.4 μg L^−1^, median = 7.8 μg L^−1^) were significantly lower than that for the neutral lake group (*p* = 0.034, by Welch Two Sample test), but not significantly different (*p* = 0.43, Mann–Whitney Wilcoxon test) from the acidified lake group (mean = 11.0 μg L^−1^, median = 8.4 μg L^−1^). Mean TP concentration for the acidified lakes was lower when compared to the neutral lake group, but the difference was not significant (*p* = 0.43, Mann–Whitney Wilcoxon test). Both the acidic and limed lake groups had individual lake median TP concentrations that were lower than the 25th and in many cases the 10th percentile values for background P concentrations estimated for lakes in southern Sweden (Huser and Fölster 2013). The naturally acidic lakes had higher TP concentrations (mean and median = 17.0 and 14.3 μg L^−1^, respectively) than all other groups but were not included in the statistical analysis due to the low number of lakes (*N* = 4) in the group.

**Fig. 3:**
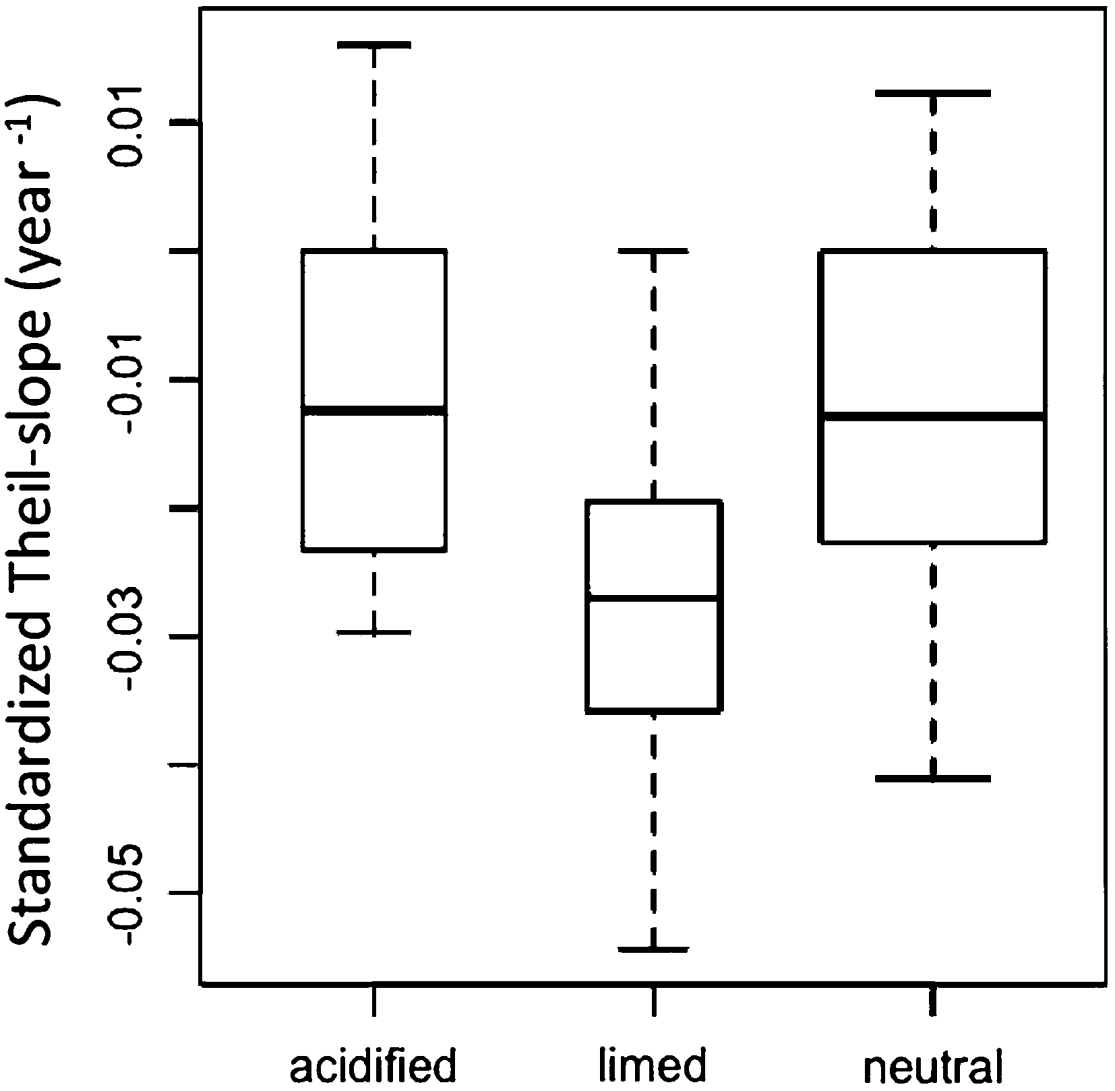
Box-plot of standardized Theil slope values (delta ug TP L^−1^ yr^−1^/mean epilimnetic TP) by lake group. In *each box*, the *central line* is the median value, the *upper* and *lower limits* of *each box* are the lower and upper quartiles (25 and 75 %), and the *whiskers* extend 1.5 times the interquartile range

Epilimnetic TP trends from 1990 to 2012 varied substantially among lake groups (Table 2). In all lake groups, the average TP concentration exhibited decreasing time trends to some extent; however, the percentage of significantly decreasing trends in each group was different, especially when considering the limed lake group. The acidified lake group had the lowest percentage of lakes with decreasing TP trends (47 %), including one lake with an increasing trend. Nearly all limed lakes had decreasing trends (85 %), whereas the percentage of decreasing TP trends in the neutral group (49 %) was similar to the acidified lake group in terms of decreasing trend percentage. The strength of decreasing TP trends also varied among groups (Fig. 3). Limed lakes had significantly (Student’s *T* test) stronger decreasing trends (standardized Theil slope mean = −0.027 μg P L^−1^ yr^−1^) than both the acidified (*p* < 0.01, standardized Theil slope mean = −0.010 μg P L^−1^ yr^−1^) and neutral lake group (*p* < 0.01, standardized Theil slope mean = −0.014 μg P L^−1^ yr^−1^), whereas the decreasing trends of the neutral lake group showed no statistical difference from the acidified lake group (*p* = 0.43). The naturally acidic lake group had no significant trends for TP concentration.

**Table 2:** Summary of statistically significant trends for epilimnetic TP (1990–2012) and Mann–Kendall Tau results of annual PRF-value index by lake group

Lake groups	No. of lakes	Increasing trend	Decreasing trend	No. trend	Increasing percentage	Decreasing percentage	Annual PRF-value index Mann–Kendall	
							Tau	*p* value
Acidified	15	1	7	7	7	47	−0.624	<0.001
Limed	13	0	11	2	0	85	−0.161	0.31
Neutral	37	0	18	19	0	49	−0.222	0.16

**Fig. 4:**
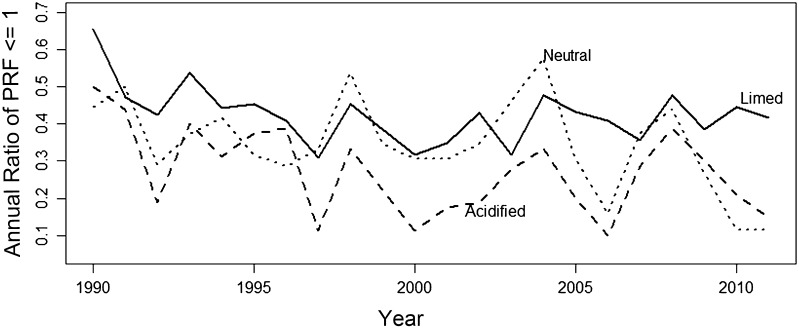
Changes in annual PRF-value index, by lake group, calculated from PRF-values from 1990 to 2011. PRF is the P release factor (i.e., hypolimnetic TP/epilimnetic TP)

**Fig. 5:**
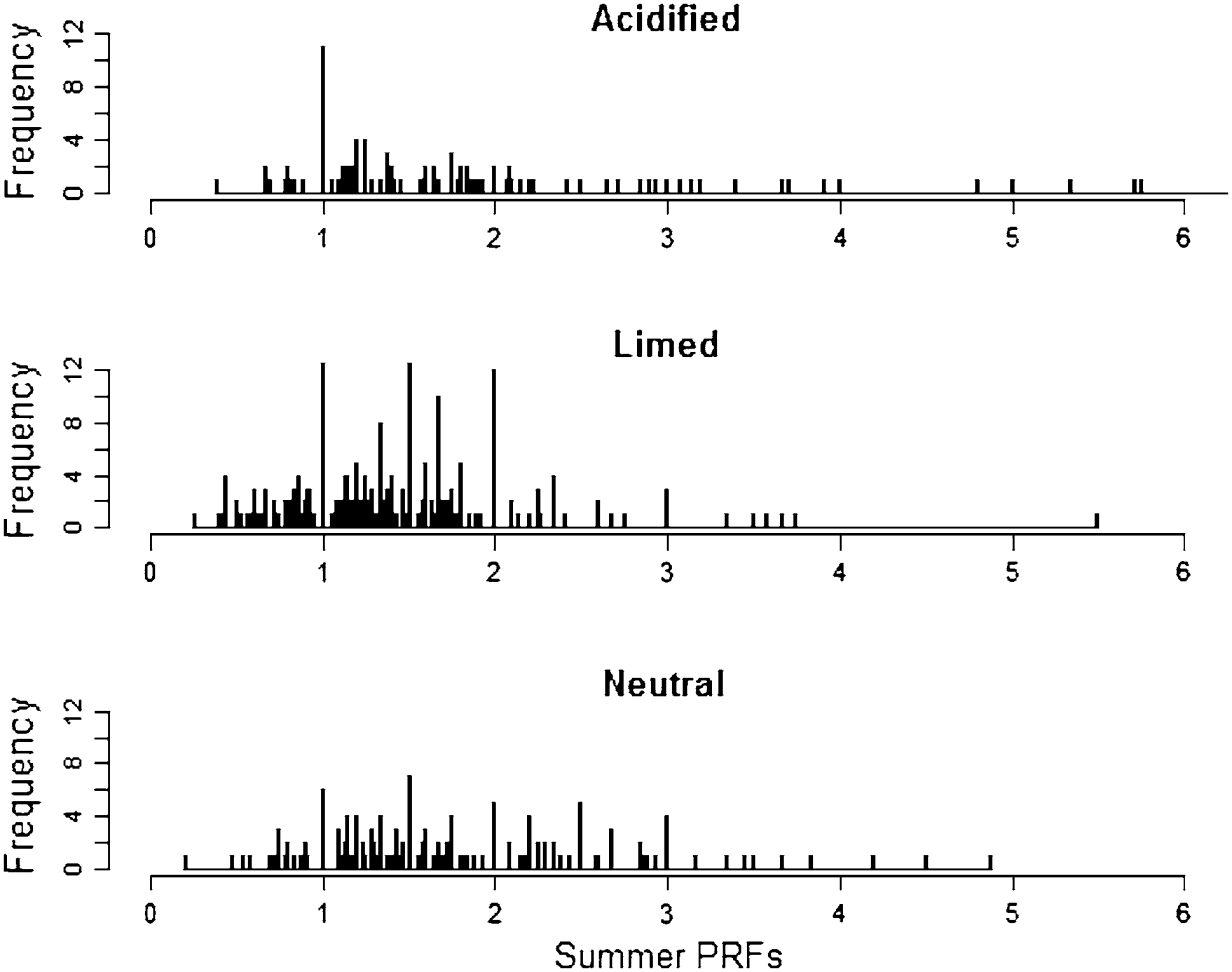
Frequency of PRF (hypolimnetic TP/epilimnetic TP)-values by lake group from 1990 to 2011. Summer is defined as the period from June to the first half of September. Only one PRF-value was used for each summer according to monthly data priority (Table 10.1007/s13280-014-0573-0), thus the prioritized months (June and July) carry more weight

The annual P release potential (annual percentage of PRF-values ≤1) for limed and neutral lakes remained more or less constant, i.e., with average PRF-values ≤1 between 30 and 40 % during 20 years of monitoring (Fig. 4). The annual percentage of PRF-values ≤1 decreased significantly over time, however, for the acidified lakes (Fig. 4), as confirmed by the Mann–Kendall test (Table 2). Summer PRF-values differed among lake groups as well, with limed lakes having a significantly higher frequency of low (≤1) PRF-values when compared to either the neutral or acidified lake groups (Fig. 5). The neutral lake group, interestingly, had a significantly greater frequency of low PRF-values than the acidified lakes. These results were confirmed by Kolmogorov–Smirnov tests (Table 3).

**Table 3:** Kolmogorov–Smirnov test results for comparison of the distributions of summer PRF-values by lake group. Interpretation: “acidified below limed” means that the frequency of relatively larger PRF-values in the acidified group was higher than that of limed lakes or the frequency of relatively small PRF-values in the acidified group was lower than that of limed group

Lake type comparison	*D* value (chosen null hypothesis)	*p* value
Acidified versus limed	*D* ^*−*^ = 0.2977, “acidified below limed”	<0.001
Acidified versus neutral	*D* ^*−*^ = 0.1387, “acidified below neutral”	<0.001
Neutral versus limed	*D* ^*−*^ = 0.1976, “neutral below limed”	<0.0001

“*D* ^*−*^” is the test statistic for Kolmogorov–Smirnov test. Visually it is the maximum distance between two curves representing the cumulative distribution of tested samples

## Discussion

Thousands of acidified lakes in Sweden have been limed, once every few years, to maintain a pH ≥ 6.0 and an alkalinity ≥0.1 meq L^−1^ (Henrikson and Brodin 1995). Because most of the treated lakes have been limed via surface applications, the tributary watersheds remain acidified, meaning the processes that limit P and elevate Al and Fe transport to lakes continue as they did previous to the start of liming activities. Persson and Broberg (1985) hypothesized that P concentrations would continue to decline in lakes with tributary watershed that remained acidified. Only 37 % of the acidified lake group, however, had negative TP trends, and they were not significantly different from the neutral lake group (Fig. 3). This result may be due to gradual recovery in some acidified lakes (and tributary watersheds that are metal sources to lakes) that began approximately a decade after regulatory controls decreased the amount of sulfur input to the atmosphere (Skjelkvåle et al. 2007). On the contrary, nearly all limed lakes had negative TP trends, even though they primarily are located in the same general region as the non-limed lakes (Fig. 1). Thus, some processes associated with liming may limit P availability in these lakes. The fact that limed lakes exhibited both a greater percentage of negative TP trends and significantly stronger trends compared with non-limed lakes (Fig. 3) indicates exacerbation (or at least a prolongation) of the oligotrophication previously detected in untreated acidified lakes.

### Metal precipitation and P internal cycling

Before large-scale liming activities were initiated, earlier research argued that P concentrations in acidified lakes were lower compared to unaffected lakes (neutral group) due to reduced inputs of terrestrial phosphorous and inhibited biological P cycling (Persson and Broberg 1985; Jansson et al. 1986; Wilander et al. 1995). Recent research has pointed to increased phosphorous sediment retention capacity caused mainly by in-lake aluminum precipitation as a potential driver for lower P concentrations (Kopacek et al. 2000; Ulrich and Pöthig 2000; Kopacek et al. 2001; Huser and Rydin 2005). Liming was expected to increase P availability in acidified lakes because an increase in pH could mitigate the negative effect on biological process favoring in-lake phosphorous cycling (Wilander et al. 1995). For instance, toxic effects on utilization of phosphate by phytoplankton would be eased because Al, which competes with phosphatases for binding sites on phosphate substances, decreased in the water column after lime treatment (Jansson et al. 1986). In addition, decomposition of organic material would also improve due to increased microbial activity and recovery of shredders (Gahnström 1995).

The biological processes that may lead to increased P cycling and availability in limed lakes, however, may not be able to offset increases in P deposition/precipitation and sediment retention capacity caused by increased precipitation and accumulation of Al, Fe, Mn due to extended formation of oxyhydroxides (Andersen and Pempkowiak 1999) as a consequence of the increase in water pH. Increased sedimentation rates have been shown in acidified lakes after liming (Andersen and Pempkowiak 1999), and elevated sediment metal burdens (Al, Fe, Mn, and other trace metals) in limed lakes have been reported (Wällstedt and Borg 2005). Furthermore, increased P accumulation rates have been tied to increasing accumulation rates for metals like Al (Huser and Rydin 2005) or both Al and Fe (Wilson et al. 2008) in the sediment of acidified lakes. Although metal precipitation and associated P burial mentioned in the above studies occurred in acidified lakes, the process can continue in limed lake as well because particles coated with Al, Fe, and Mn have great affinity for phosphorous (Froelich 1988), and metal precipitation processes are enhanced by the elevated pH (generally between 6 and 7) often present limed lakes. Al is especially important with respect to P cycling because Al-oxyhydroxides are not sensitive to changes of redox potential, unlike similar Fe forms (Kopacek et al. 2000; Ulrich and Pöthig 2000; Kopacek et al. 2001). Negligible amounts of P have been shown to be released during hypolimnetic anoxia when the ratio of sediment Al to Fe (or P) is above a certain level (Kopacek et al. 2005; Wilson et al. 2008). High amounts of Fe, however, can form Fe–P complexes with multidentate ligands (Stumm and Morgan 1996) with high stability, which also might prevent P release from sediment.

In an attempt to determine if differences in epilimnetic P concentrations could be related to P release from sediment and availability in bottom waters, we developed the PRF, which can indicate a lack of P cycling from the sediment when the PRF-ratio is ≤1. Cycling of P between sediment and water is a common and natural process, even in oligotrophic systems. During stratification periods, P is often elevated in the hypolimnion compared to the epilimnion due to a combination of settling particles (both organic and inorganic), release of sediment P from Fe and Mn when bottom waters become anoxic, and release of P from mineralized sediment organic matter. The above processes are generally strongest during the growing season when temperatures are higher (elevated microbial activity) and lakes are stratified, thereby limiting oxygen penetration to the hypolimnetic zone. Thus, from a seasonal perspective, higher PRF-values are likely to be detected during the growing season (i.e., May to September in southern Sweden).

A comparison of PRF-values among lake groups suggests internal P cycling as a limiting factor in limed lakes compared with non-limed lakes, at least during the growing season when biological nutrient demand is at its highest. Between 1990 and 2011, there was a significantly higher frequency of occurrence of summer PRF-values between 0 and 1 in the limed lake group compared with the acidified and neutral lake groups (Fig. 5), indicating lower potential for internal P cycling in limed lakes. The annual PRF-value index (annual percentage of PRF-values ≤1) for the limed lake group was generally high and consistent (≈42 %), whereas the acidified and neutral lake groups showed significant (*p* < 0.001) and non-significant (*p* = 0.16) decreasing index trends, respectively (Fig. 4). Thus, the non-limed lake groups generally had an increasing tendency for internal P cycling (likely due in part to chemical recovery from acidification as mentioned above), a tendency not found in the limed lake group. The restricted internal P cycling in the limed lake group coincides with lower mean epilimnetic TP values and stronger decreasing trends for TP concentration, suggesting that reduced P release from sediment may limit overall epilimnetic P availability and potentially productivity in limed lakes.

Constrained internal P cycling can likely be attributed to enhanced metal precipitation due to increases in lake water pH, photo-oxidation and release of Fe and Al from organic matter, and P binding metals (Fe and Al) contained in liming materials (Huser and Rydin 2005; Wällstedt and Borg 2005; Kopacek et al. 2007). A previous investigation from 1989 to 1998 in the ISELAW program supports this conclusion, wherein 13 continuously limed lakes had lower trophic status and reduced productivity compared to neutral reference lakes (Appelberg and Svenson 2001; Persson and Appelberg 2001). Likely the biological recovery in many of the limed lakes included in a review by Clair and Hindar (2005) may be due to nutrient limitation in some of the lakes. Unfortunately, the information needed to quantitatively verify the above hypothesis (e.g., hypolimnetic P concentration and sediment data) is lacking from most studies.

### Other factors that may affect P concentrations

It was unexpected that the acidified and neutral lake groups in our study were not statistically different with respect to epilimnetic P concentrations. Variable trends in color, or TOC, between limed and non-limed lake groups might be one explanatory factor. Analysis of TOC data from 1990 to 2012 showed that almost all lakes had increasing trends (data not shown), and acidified lakes had stronger increasing TOC trends than limed and neutral lakes (Electronic Supplementary Material, Fig. 10.1007/s13280-014-0573-0). It should be noted that long-term data for DOC were not available for use in our study, but DOC generally constitutes [“approximate” symbol here] 90 % of TOC in Swedish surface waters (Köhler 2010), and increasing water color (and/or TOC) in Swedish and Norwegian surface waters has been previously reported (e.g., Hongve et al. 2004; Huser et al. 2011). DOC concentrations have been shown to increase (Evans et al. 2005; Skjelkvåle et al. 2005; Monteith et al. 2007) and correlate positively with P (Kopacek et al. 2006) in acidified lakes recovering from acidification. DOC leaching from watershed soils increases as acidity decreases, thus decreasing sulfate deposition may accelerate terrestrial carbon loss (Evans et al. 2012). Decreasing sulfate concentrations have been shown across the northern hemisphere, including Sweden (Garmo et al. 2014), and these trends are generally consistent on a regional basis (both deposition and in-lake concentration). In addition, P transported to lakes from drainage areas is generally associated with humic substances (Ulrich and Pöthig 2000). Because the pH in acidified, non-limed systems has been generally increasing due to reduction in deposition of acidifying compounds (and thus lower the ionic strength in the water), an increase in DOC would be expected. The higher increasing trends for TOC from 1990 to 2012 found for the acidified lakes in our study (Fig. 10.1007/s13280-014-0573-0) may partly explain why TP concentrations in the acidified and neutral lake groups were not significantly different during this period, even though acidified lakes have been previously shown to have lower TP concentrations than similar lakes not altered by acid deposition (Persson and Broberg 1985; Jansson et al. 1986; Wilander et al. 1995; Kopacek et al. 2000; Ulrich and Pöthig 2000; Kopacek et al. 2001).

Fe may also partially explain a significant share of variation in water color in terms of TOC in Swedish waters because increasing iron has been shown to associate with organic matter which is of terrestrial origin (Huser et al. 2011; Kritzberg and Ekström 2011). Because dissolution and transport processes occurring in watersheds of acidified and limed lakes should be similar with respect to recovery and changes in organic matter transport, it may be that the increased transport of TOC/DOC to acidified and limed systems is being precipitated in limed systems due to metal precipitation induced by elevated pH (Wällstedt et al. 2009), especially that bound to Fe (Köhler et al. 2014). The neutral pH in the limed and neutral lakes likely causes organic carbon to act in a similar manner chemically with respect to in-lake concentrations of TOC (i.e., a smaller proportion remains in dissolved form), which is supported by similar trends for these two groups shown in Fig. 10.1007/s13280-014-0573-0.

Besides the altered metal precipitation and differences in dissolved organic matter detected in the study lakes, other factors such as lake geomorphology, catchment characteristics, and climatic factors can affect phosphorous dynamics in lakes (Hupfer and Lewandowski 2008; Persson and Broberg 1985). Because the above factors did not vary substantially between the lake groups (less than a factor of two, Table 1), we have omitted these factors as explanatory variables for the variation in P trends between lake groups. Future work, however, could include a closer look at how factors such as climate and lake geomorphology affect P availability and biological recovery in acidified and limed lakes.

## Conclusions

Limed lakes had lower TP concentrations than non-limed lakes (although only significant compared to neutral lakes), likely due in part to limited cycling of P between sediment and water.

P cycling between sediment and water in limed lakes seems constrained by elevated metal concentrations (i.e., Fe and Al) in sediments, as they can act as precipitators/co-precipitators for P. Because P is often the limiting growth factor in freshwater systems, limed lakes, which naturally often have low P levels, might be severely affected.

The unexpected similarity in TP concentrations found between the acidified and neutral lake groups may be associated with increased dissolved organic matter concentrations, but more work is needed to elucidate the reason(s) behind this phenomenon.

## Electronic supplementary material

Below is the link to the electronic supplementary material.

Supplementary material 1 (PDF 397 kb)
