# Supplementary material for: Anthropogenic oligotrophication via liming: Long-term phosphorus trends in acidified, limed, and neutral reference lakes in Sweden
Source: Ambio. 2014 Nov 15;43(Suppl 1):104–12. doi: 10.1007/s13280-014-0573-0 (PMC4235932; doi:10.1007/s13280-014-0573-0)
Supplement: Supplementary file 1 — Supplementary material 1 (PDF 397 kb) [file 13280_2014_573_MOESM1_ESM.pdf]

AMBIO

Electronic Supplementary Material

This supplementary material has not been peer reviewed.

Title: Anthropogenic oligotrophication via liming: Long-term phosphorus trends in acidified, limed and neutral reference lakes in Sweden

Authors: Qian Hu, Brian Huser

Table S1 Seasonal divisions and monthly data priorities for the study lakes

**Season**	**Months included**	**First rank**	**Second rank**	**Third rank**	**Forth rank**
Winter	December (previous year), January, February, March	March	February	January	December (previous year)
Spring	April, May	April	May		
Summer	June, July, August, First half of September	August	July	June	First half of September
Autumn	Second half of September, October, November	October	November	Second half September	

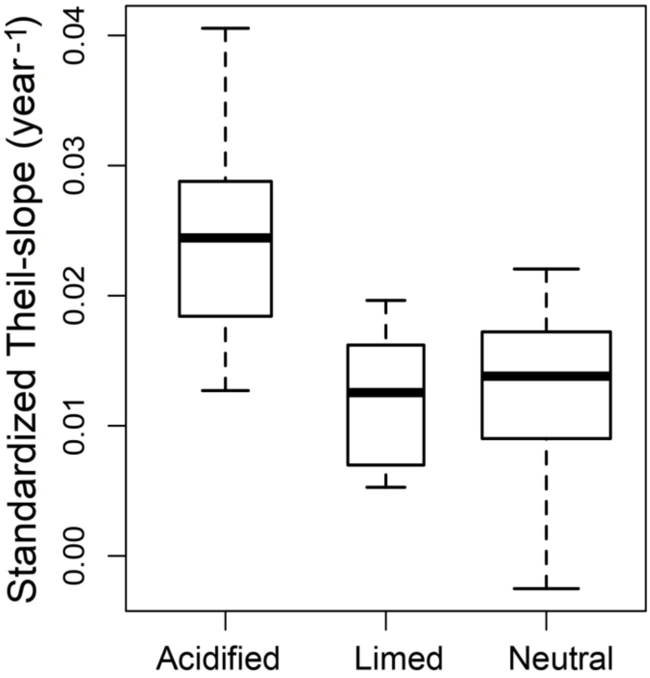

Fig. S1 Box-plot of standardized Theil-slope values (delta mg TOC L^{-1} yr^{-1}/mean epilimnetic TOC) by lake group. In each box, the center line is the median value, the upper and lower limits of each box are the lower and upper quartiles (25% and 75%), and the whiskers extend 1.5 times the interquartile range
